# Review of Professor Peirce’s Paper, “Pyorrhœa Alveolaris”

**Published:** 1894-04

**Authors:** R. R. Andrews

**Affiliations:** Boston, Mass.


### REVIEW OF PROFESSOR PEIRCE'S PAPER, "PYORRHŒA ALVEOLARIS."[1]

BY DR. R. R. ANDREWS, BOSTON, MASS.

Your letter of February 8, asking me for my views regarding Professor Peirce's article on "Pyorrhœa Alveolaris," published in the January number of the International Dental Journal, has been received. I hardly know what reply I should make. I do not profess to be a specialist on this subject, although I am greatly interested in it. Recently I have given some attention to the microscopical examination of sections of roots of teeth lost by reason of this disease. I wish to study carefully the very marked change in the character of the dentine of the root, and I should be obliged to any member of your Society who would aid me in this investigation. If they can send me teeth which have been lost from this cause, in diluted alcohol, placing them in this just after extraction, before they have become dry, together with a short history of the case, it would be quite a help to me. I have made a number of sections of these teeth, but want more for comparison. We are to be congratulated that at last we have been thoroughly aroused to the importance of knowing more about this most perplexing disease, "pyorrhœa." Indeed, Professor Peirce's article is claiming the attention of the whole profession, and this must necessarily give us results which will lead up to a more successful treatment. I do not know how many cases of true serumal tartar from different teeth, taken from the apices of the roots, have been carefully analyzed. If the number should be large, and all show traces

---

[1] Read before the Eighth District Dental Society, Buffalo, N. Y., February 26, 1894.

of uric acid and its salts, it must necessarily go a great way towards settling this theory. The tartar which we find on the palatal portion of the upper incisors just under the gum and on the palatal roots of the molars in the mouths of comparatively young people I have always considered to be one form of true serumal tartar. Some of our oldest and best operators make the statement that patients of whom they have the charge from youth up never contract this disease, although their parents may have lost their teeth by it. Proper care at the proper time has prevented its occurrence. These men are loath to believe that this tartar is a deposit from the blood,—being deposited before pockets are formed. Gouty and rheumatic people among my own patients have not, as a rule, been more troubled with this disease than others. I have not given the uric-acid theory—advanced before Professor Pierce came on the field—much consideration, but the analysis showing the actual presence of uric acid in this deep-seated tartar demands serious consideration and careful investigation. If we go at this work in the same careful manner as that pursued by the worthy president of your State Society while investigating this subject, we should soon settle the question whether concretions of serumal tartar can exist upon or near the apices of roots of teeth, without a break in the continuity of the gum tissue at its gingival border. I have held there could be no deposit, not even of the nodular tartar, without an inlet from the gum to its location. This is one of the important things that we must prove, and Dr. Van Woert's method is the only proper way to investigate. We must look for its solution at dissecting tables and at autopsies. We must interest the students of the dissecting-room, both dental and medical, and before a great while we shall have facts that will give us the exact truth.

Another question arises. Shall it be possible, by any system of medication, to dissolve this deposit from the roots of teeth, in its earlier stages, before the pockets are formed, while yet there is more or less irritation? I do not believe this to be possible. I have been led to believe, from my own experience, that this trouble exists largely in the mouths of people accustomed to luxury,—good livers, people above middle age, who over-eat and under-work. If intelligent medication, with the end in view of eliminating uric acid from the system, and with the proper diet for preventing its formation, shall be followed by a cessation of the symptoms and the trouble itself, it must necessarily prove the accuracy of Professor Peirce's hypothesis. Enough has been proved to make it the duty of every conscientious operator to carefully carry out this scheme of medication, and after proper time report the results to his society. All the care that has been given to the removal of tartar from the roots heretofore, together with the thorough use of antiseptics, must be continued with the additional medication suggested. This theory of Professor Peirce appeals to every one of us; it sounds rational; it seems the most plausible solution of this difficult problem that has been offered.

Professor Peirce calls our attention to one very important fact, and that is, that disturbed functional activity causes the deposition of the salts of uric acid in the tissue of the pericementum, and any undue irritation, even the simple wedging of teeth, may start up this trouble. If this danger signal shall stop many of the younger members of our profession, and some of the older ones too, from the excessive use of the wedge, it will prove a great blessing to suffering humanity, and perhaps save their teeth from "hæmatogenic calcic pericementitis."

---

